# A step toward diagnostic reference levels for male varicocele embolization: dosimetric comparison across two generations of angiographic systems

**DOI:** 10.1007/s11547-025-02163-z

**Published:** 2026-01-08

**Authors:** Laura Maria Cacioppa, Francesco Mariotti, Alberto Mari, Alessandra Bruno, Nicolò Rossini, Giangabriele Francavilla, Marco Macchini, Marzia Rosati, Davide Matteagi, Paolo Veccia, Edoardo Bindi, Andrea Benedetto Galosi, Giovanni Cobellis, Roberto Candelari, Chiara Floridi

**Affiliations:** 1 Division of Interventional Radiology, Department of Radiological Sciences, University Politecnica Delle Marche, Ancona, Italy; 2 Department of Clinical, Special and Dental Sciences, University Politecnica Delle Marche, Ancona, Italy; 3 Division of Medical Physics, Department of Radiological Sciences, University Hospital “Azienda Ospedaliero Universitaria Delle Marche”, Ancona, Italy; 4 Pediatric Surgery Unit, Salesi Children’s Hospital, University Hospital “Azienda Ospedaliero Universitaria Delle Marche”, Ancona, Italy; 5 Urology Unit, University Hospital “Azienda Ospedaliero Universitaria Delle Marche”, Ancona, Italy; 6 Division of Radiology, Department of Radiological Sciences, University Hospital “Azienda Ospedaliero Universitaria Delle Marche”, Ancona, Italy

**Keywords:** Dose area product, Fluoroscopy time, Radiation dose, Radiation protection, Varicocele, Therapeutic embolization, Male subfertility

## Abstract

**Purpose:**

Percutaneous endovascular embolization (PVE) of male varicocele is a widely adopted procedure performed in healthy young patients with long life expectancy. Dose-optimization systems are therefore essential to minimize procedural radiation risks. We aimed to investigate the effect of a recently implemented dose-reduction technology on dosimetric parameters and its potential implication in the definition of diagnostic reference levels (DRLs).

**Materials and Methods:**

A consecutive series of 113 patients (23.3 ± 9.1 yrs) submitted to PVE between January 2020 and December 2024 were retrospectively reviewed. Two groups based on the angiographic system used, 50 patients treated using the Philips Allura Xper FD20 and 63 patients using the newer Philips Azurion Clarity IQ technology, were compared in terms of demographic, procedural, and dosimetric data.

**Results:**

Despite similar fluoroscopy and procedure times, the recently implemented angiographic technology demonstrated a significant reduction in dose area product (DAP) and reference air kerma (Ka.ref) values (4394.5 vs 20,709 mGy·cm^2^ and 20.35 vs 83.6 mGy; *p* < 0.001, respectively) with a percentage reduction of approximately 71.15% and 64.41%, respectively. Subpopulation analyses showed significant dose reductions in younger patient population (< 18 years) and in high-grade varicoceles (grades III/IV), with similar fluoroscopy times.

**Conclusion:**

The use of advanced dose-optimization technologies, together with standardized protocols and appropriate operator training, leads to a significant reduction in radiation exposure during PVE, particularly in younger patients. These single-center, retrospective results provide preliminary data that may support the future introduction of procedure-specific DRLs for this routinely and widely performed interventional radiology procedure.

## Introduction

Varicocele is a common condition occurring in healthy young male patients defined as an abnormal dilation of pampiniform plexus veins due to venous reflux [1, 2]. Approximately 80–90% of cases affect the left side, while 5–10% the right side and 1–15% is bilateral [3, 4]. Although commonly asymptomatic, it can cause discomfort or even scrotal pain and impaired spermatogenesis [5]. According to current guidelines, indications for treatment are palpable or painful varicocele, reverse or prevention of testicular atrophy, and male infertility with abnormal sperm quality [4–6]. Over the past two decades, percutaneous endovascular embolization (PVE) has widely become the first treatment option due to the undeniable advantages of being an outpatient procedure with testicular artery sparing, a faster recovery, reduced complications, lower costs and recurrence rates [7]. On the other hand, interventional procedures are often demanding in terms of radiation exposure, with increased risk of stochastic effects for both patients and staff [8]. Several factors affect radiation doses during PVE including operator experience, fluoroscopy time, procedure complexity, the conscientious use of dose-reduction methods and the quality of equipment. Since the procedure is performed in healthy young males and is also eligible for effective surgical alternatives, dose reduction to the minimum achievable is of paramount importance, as recommended by the International Commission on Radiological Protection (ICRP) [8, 9]. In addition, EURATOM Council Directive 2013/59 and its Italian transposition (Legislative Decree 101/2020) have strengthened the role of the diagnostic reference levels (DRLs) in facilitating dose optimization in several interventional radiology practices [10–14]. DRLs are dose levels for typical examinations of standard-sized patients established using specific equipments. DRLs have been introduced to optimize radiation protection, should not be used as dose limits and may be exceeded when clinically justified. However, consistently exceeding DRLs suggests that radiation doses are regularly above national reference standards, indicating a need for review and possible corrective measures. One of the methods commonly used to establish DRLs is based on collecting data from a group of patients representative of population and calculating the 75th percentile of the dose distribution [15, 16]. This value may be considered as the DRL for a defined population undergoing a specific radiological examination and imaging facility. Although being a largely performed procedure, determining DRLs for PVE is particularly challenging given the wide distribution of doses within the same procedure due to vascular anatomical variants, different access sites and embolic agents, and the influence of procedure complexity [11–13, 17].

The most recent angiographic systems have contributed to the efforts of dose optimization introducing technological developments such as low-dose fluoroscopy, lower pulse rates, virtual collimation and virtual patient positioning to improve image quality minimizing radiation doses [11, 18].

This study aimed to evaluate the impact of the recently installed optimized technology on radiation doses delivered during PVE procedures in our center, providing preliminary insights relevant to the future definition of procedure-specific DRLs for varicocele treatment.

## Materials and methods

### Study design and patient population

This study received ethics approval from the Ethics Committee of our Institution and was conducted in conformity to the ethics guidelines of the 2024 Declaration of Helsinki and its amendments. All patients provided an informed written consent to the procedure. For pediatric patients, parental consent was obtained.

We performed a single-center retrospective analysis of data collected from 113 young males affected by varicocele referred to the Urology and Pediatric Surgery outpatient services and treated in the Interventional Radiology of our tertiary referral center between January 2020 and December 2024.

Patients were intentionally enrolled as follows:

- 50/113 patients consecutively treated between January 2020 and September 2022 (group 0)
- 63/113 patients consecutively treated between November 2023 and December 2024 (group 1)

Demographic and clinical data were obtained from electronic medical records. Files and images were extracted from RIS (Radiology Information) and PACS (Picture Archiving and Communication Systems—GE Medical System, Milwaukee) of the hospital.

Demographics, anatomical, clinical and procedural characteristics, and dosimetric data were collected in a dedicated database. Age, presence of primitive or recurrent varicocele, grade of varicocele according to the ultrasound classification of Sarteschi, presence of clinical symptoms including testicular pain or discomfort, and sperm count anomalies (in adolescents or young adults) were recorded. BMI and body habitus were not included, as they were not required for the dosimetric comparisons. Procedural parameters included left spermatic vein type according to the Bahren angiographic classification, average procedural time, embolic agent (mechanical, liquid, or a combination of both) and number of microcoils employed.

#### Inclusion criteria and treatment indications

Inclusion criteria included:

- Male patients diagnosed with left-sided varicocele during urological examination in our department, including anamnestic review, physical examination and duplex ultrasound (DUS), to assess testicular volume, parenchymal structure and vascularity, and the presence, extent and degree of pathological venous reflux at rest and after the Valsalva maneuver.
- Fully completed and standardized PVE procedures with complete available dosimetric data performed at our Interventional Radiology Unit.

Exclusion criteria included right-sided or bilateral varicocele treatments, due to potentially longer procedural and fluoroscopy times and increased technical complexity.

Indications for treatment were from the European Cardiovascular and Interventional Radiological Society Standards of Practice [4].

#### Procedural characteristics

All the procedures were performed in outpatient setting by one of the six institutional interventional radiologists each having at least 5 years of experience. The procedures were performed under local anesthesia (10 mL, Lidocaine 1%), after skin disinfection and preparation of the sterile field. A right transfemoral US-assisted venous approach was used in all cases. After insertion of a 4-F vascular sheath, a 4-F hydrophilic Cobra catheter (Cordis Inc. Bridgewater, NJ) preloaded with a 180-cm 0.035-inch angled hydrophilic guidewire (Terumo Europe, Leuven, Belgium) was introduced into the sheath and guided fluoroscopically to the inferior vena cava (IVC) and the left renal vein (LRV) orifice. Selective catheterization of the left internal spermatic vein (ISV) was then achieved using either the hydrophilic guidewire. If the spermatic vein could not be cannulated after certain attempts, the catheter tip was used to identify the spermatic vein orifice through the injection of a small amount of contrast medium. Once catheterized, spermatic vein course, size, collateral pathways, vein type and variations according to Bahren classification were assessed by a firm manual injection of 5–10 mL of contrast medium during Valsalva maneuver. Left renal venography was limited to in very rare cases of suspected anatomical variations of IVC or LRV. After a microcatheter-microguide systems 2.4 or 2.7 Fr (Progreat, Terumo Medical, Tokyo, Japan or Drakon Microcatheter, Guerbet, Roissy, France), distal extremity was coaxially advanced at the level of the inferior part of sacroiliac joint, the ISV was embolized by releasing pushable 0.018" platinum microcoils (Boston scientific, Natick, MA, USA) in variable number and oversized by 2–3 mm. All the procedures were completed by the injection of 2–6 ml 2% polidocanol (2% Aethoxysklerol, Chemische Fabrik Kreussler & Co. GmbH, Wiesbaden), foamed with 2 cc of contrast medium and 2 cc of air, into the ISV under fluoroscopic vision and fractionated into 2–3 mL boluses until reaching a stable contrast medium column and avoiding reflux to the LRV. During the injection, an external compression of the vein over the pubic bone, or a tourniquet application at the base of the scrotum was performed and maintained for the following 1–2 min. Lastly, the catheter was withdrawn to the proximal ISV and contrast injected to document occlusion. During the procedure, the interventionalist could decide the feasibility of embolizing collateral and aberrantly fed veins. Hemostasis of the puncture site was obtained by manual compression.

As reported by CIRSE guidelines [4], all patients had bed rest in the day unit for the first 3–4 h after the procedure and then discharged with the indication to rest completely for the next 24 h. All patients underwent a routine clinical visit one week post-procedurally and were instructed to avoid physical exertion and sexual activity for at least 3 weeks, to report potential complications, and to treat post-procedural pain with a non-steroidal anti-inflammatory drugs or paracetamol.

All the procedures were performed in two fully equipped angiosuites of our institution:

- Group 0 underwent PVE using Philips Allura Xper room with FD 20 technology (Philips Healthcare, Best, Netherlands) (System 0);
- Group 1 underwent PVE using the newest Philips Azurion 7 M20 with Clarity IQ technology (Philips Healthcare, Best, Netherlands) (System 1).

All PVEs were performed with the C-arm image intensifier placed as close as possible to the patient and used in automatic modality with a frame rate of 7.5 pulses/s, and the X-ray tube placed in undercouch position. A standardized protocol aimed to minimize radiation doses was adopted. The protocol consisted in maintaining fluoroscopy time and mode as low as possible, adjusting field collimation, preferring pulsed fluoroscopy, avoiding oblique projections, angiographic runs magnifications and duplicative images.

#### Radiation exposure measurements

Radiation exposure during PVE was assessed by means of the following dosimetric parameters: fluoroscopy time (FT, min); dose area product (DAP, mGy·cm^2^) or air-area kerma product (PKA), and reference air kerma (Ka,ref; mGy). Definitions were from the Society of Interventional Radiology quality improvement guidelines for recording patient radiation dose [19]. Number of series and number of images acquired in each procedure were also registered. The dosimetric measurements were recorded using a dose-detector software and archived with images. Dosimetry was analyzed stratifying the study population in two further subgroups at higher radioprotection priority: patients under 18 years of age and patients with high-grade varicocele.

#### Procedural image analysis

A retrospective qualitative assessment of procedural image was independently performed by two of our interventional radiologists with at least 5 years of experience in PVE. Each interventionalist, blinded to clinical data, procedural and dosimetric details, retrospectively assessed image sets obtained with the two angiographic systems. The assessment was based on three predefined image quality parameters, vessel delineation, contrast resolution, and anatomical detail visibility, as suggested by previous studies [20, 21]. Each parameter was scored using a 4-point Likert scale (1 = non-diagnostic, 2 = moderate but sufficient for diagnosis, 3 = good, 4 = excellent).

#### Statistical analysis

All statistical analyses were conducted by SPSS version 22 software (IBM Corp., Armonk, NY, USA). A two-tailed *p*-value of < 0.05 was considered statistically significant. Quantitative variables of the study population were reported as mean ± standard deviation (SD), and as median and range, as applicable, to account for non-normal distributions. Categorical variables were reported as numbers and percentages.

Shapiro–Wilk test was used to assess the normality of data distribution. Comparative analyses between groups were conducted using the Mann–Whitney U test for continuous non-normally distributed variables and the independent samples t-test for normally distributed data. Chi-squared test or Fisher’s exact test was applied for categorical data.

Boxplots and Violin plots were included to illustrate the distribution of key dosimetric and procedural parameters across the different angiographic systems and subpopulations. Data visualization and plot generation were performed using GraphPad Prism version X (GraphPad Software, San Diego, CA, USA).

## Results

### Demographic, clinical and procedural findings

A total of 113 PVE procedures in 113 patients were included, 50 performed with System 0 (group 0) and 63 performed with System 1 (group 1). The mean age was 23.3 ± 9.1 years (range 16–29.3 years). Patients treated with System 1 were slightly younger (median age 19 years, range 14–37) compared with those treated with System 0 (median age 22 years, range 12–37).

All the included cases had left varicocele, with a great prevalence of primitive varicocele (111/113) and two cases of recurrence after surgery. According to the Sarteschi sonographic classification, 22% of patients were diagnosed with grade II varicocele, 46% with grade III, and 32% with grade IV. The enrolled patients complained of a testicular pain in 75 (66%) of cases and infertility in 12 (11%) cases. Nineteen (17%) patients complained of both the conditions. In 7 (6%) cases, another condition such as small testis or testicular asymmetry was detected. Demographic, anatomical and clinical characteristics of the study population are summarized in Table 1.

**Table 1:** Demographic, anatomical and clinical characteristics of study population (*n* = 113)

Demographic	Total population (*n* = 113)	Group 0 (*n* = 50)	Group 1 (*n* = 63)	*p*
Age				
Median (years)	21	25.5	19	0.234
Mean (years ± SD)	23.3 ± 9.1	26	20.4 ± 7.4	
IQR (years)	16–29.25	19–31	17–27.25	
*Anatomical characteristics*				
Left varicocele	113 (100%)	50 (44%)	63 (56%)	
Bilateral varicocele	0	0	0	
Primitive varicocele	111(98.2%)	48 (42.5%)	63 (55.7%)	
*Sarteschi sonographic grade*				
Grade II	25 (22.1%)	17 (15%)	8 (7.1%)	
Grade III	52 (46%)	23 (20.4%)	29 (25.7%)	
Grade IV	36 (31.9%)	10 (8.8%)	26 (23%)	
Clinical indication*				
Recurrence	2 (1.8%)	2 (1.8%)	0 (0%)	
Testicular pain	75 (66.4%)	35 (31%)	40 (35.4%)	
Infertility	12 (10.6%)	6 (5.3%)	6 (5.3%)	
Pain and infertility	19 (16.8%)	9 (8%)	10 (8.8%)	
Other	7 (6.2%)	0 (0%)	7 (6.2%	

Group 0 refers to procedures performed using the Philips Allura Xper FD20 system; group 1 refers to procedures performed using the Philips Azurion Clarity IQ system

^*^Percentages for clinical indications may exceed 100% as some patients presented with more than one indication for treatment

Concerning procedural details, all patients were treated by transfemoral access.

According to fluoroscopic sequences obtained by manual injection of contrast medium, 64 (57%) patients were classified as Baehren type I, 7 (6%) patients had a type II and 39 (34%) patients had a type III anatomy. Types IV and V were demonstrated in 2 (2%) and 1 (1%) patients, respectively.

Spermatic vein embolization was obtained by releasing pushable microcoils of various sizes in combination with 1% aethoxysklerol injection in 112 (99%) of cases. In one case, the liquid agent was injected alone. The average number of microcoils employed in each procedure was 4.8 ± 2.4 units. Angiographic and procedural characteristics of study population are reported in Table 2.

**Table 2:** Angiographic and procedural characteristics of study population (*n* = 113). Group 0 refers to procedures performed using Philips Allura Xper FD20 system; group 1 refers to procedures performed using Philips Azurion Clarity IQ system

Angiographic features n (%)	Total population (n = 113)	Group 0 (n = 50)	Group 1 (n = 63)	*p*
*Bahren angiographic type*				
Type I	64 (57%)	30 (27%)	34 (30%)	
Type II	7 (6%)	3 (3%)	4 (4%)	
Type III	39 (34%)	14 (12%)	25 (22%)	
Type IV	2 (2%)	2 (2%)	0 (0%)	
Type V	1 (1%)	1 (1%)	0 (0%)	
*Procedural characteristics n (%)*				
Microcoils insertion	112 (99%)	49 (43%)	63 (56%)	
Liquid agent injection	113	50 (44%)	63 (56%)	
Number of microcoils/procedure	4.8 ± 2.4	3.9 ± 1.2	5.5 ± 2.8	
*Procedure time*				
Median (min)	27	25.5	28	0.23
Mean (min + SD)	29.7 ± 10.6	28.3 ± 8.7	30.7 ± 11.7	
IQR (min)	22–35	21.75–33	22–36.25	

The average procedure duration was 29.7 ± 10.6 min, with a range between 22 and 35 min.

No significant differences in terms of procedure duration were reported comparing the two groups (25.5 vs 28 min; *p* = 0.23). Group 1 demonstrated a higher variability in procedure duration.

### Dosimetric results

The mean fluoroscopy time was 656 s for group 0 and 667 s for group 1, with no statistically significant differences between the two populations (*p* = 0.41).

Dose area product (DAP) median values were: 4394.5 (IQR: 1916.25–8057.75) mGy·cm^2^ in group 1 and 20,709 (IQR: 13,481.25–41,347) mGy·cm^2^ in group 0 (p < 0.001). Reference air kerma (Ka.ref) median values were: 20.35 (IQR: 12.40–36.12) mGy in group 1 and 83.6 (54.77–131.03) mGy in group 0 (*p* < 0.001). A summary of the main dosimetric findings is provided in Table 3.

**Table 3:** Summary of the radiation exposure parameters obtained in the two groups

Outcome	Total Population	Group 0	Group 1	*p*
	(*n* = 113)	(*n* = 50)	(*n* = 63)	
*Fluoro time (sec)*				**0.41**
Median	482	446	516	
Mean	662	656	667	
IQR	355–839	301.5–644.25	365–870.5	
*Fluoro DAP (mGy·cm*^*2*^*)*				** < 0.001**
Median	9455	20,709	4394.5	
Mean	18,275	35,276	6210	
IQR	4184.75–19,312	13,481.25–41,347	1916.25–8057.75	
*Ka.ref (mGy)*				** < 0.001**
Median	38	83.6	20.35	
Mean	71.75	122.75	27.74	
IQR	18.4–88.84	54.77–131.03	12.40–36.12	

**Fig. 1:**
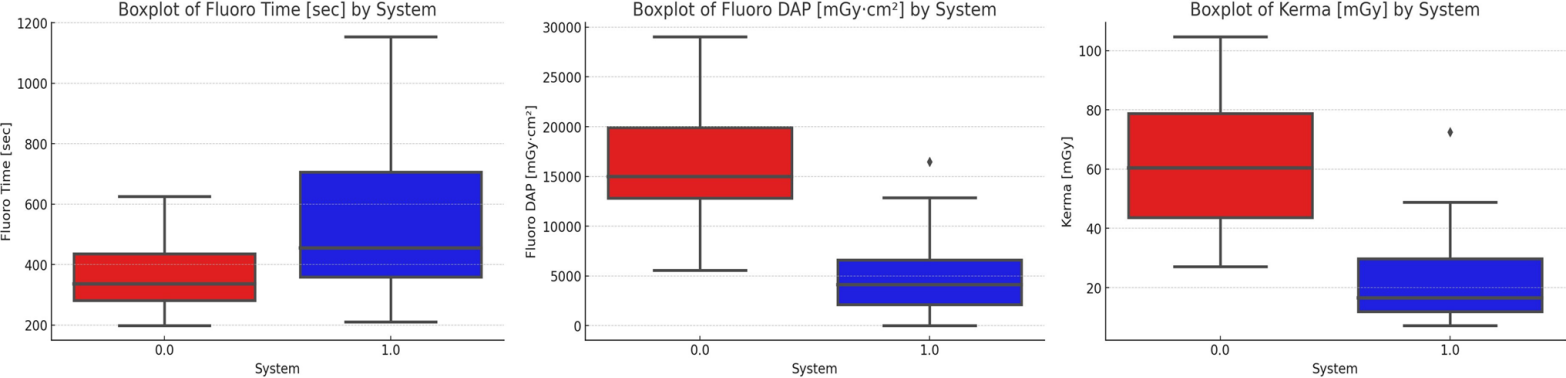
Boxplots comparing fluoro time (left), dose area product (DAP, center), and reference air kerma (right) between System 0 (red) and System 1 (blue). While fluoro time appeared higher and more variable with System 1, DAP and Kerma values were markedly lower, indicating improved radiation efficiency despite longer procedure durations

The boxplot graphs comparing fluoro time, DAP, and Ka.ref between Group 0 and Group 1 are illustrated in Fig. 1.

**Fig. 2:**
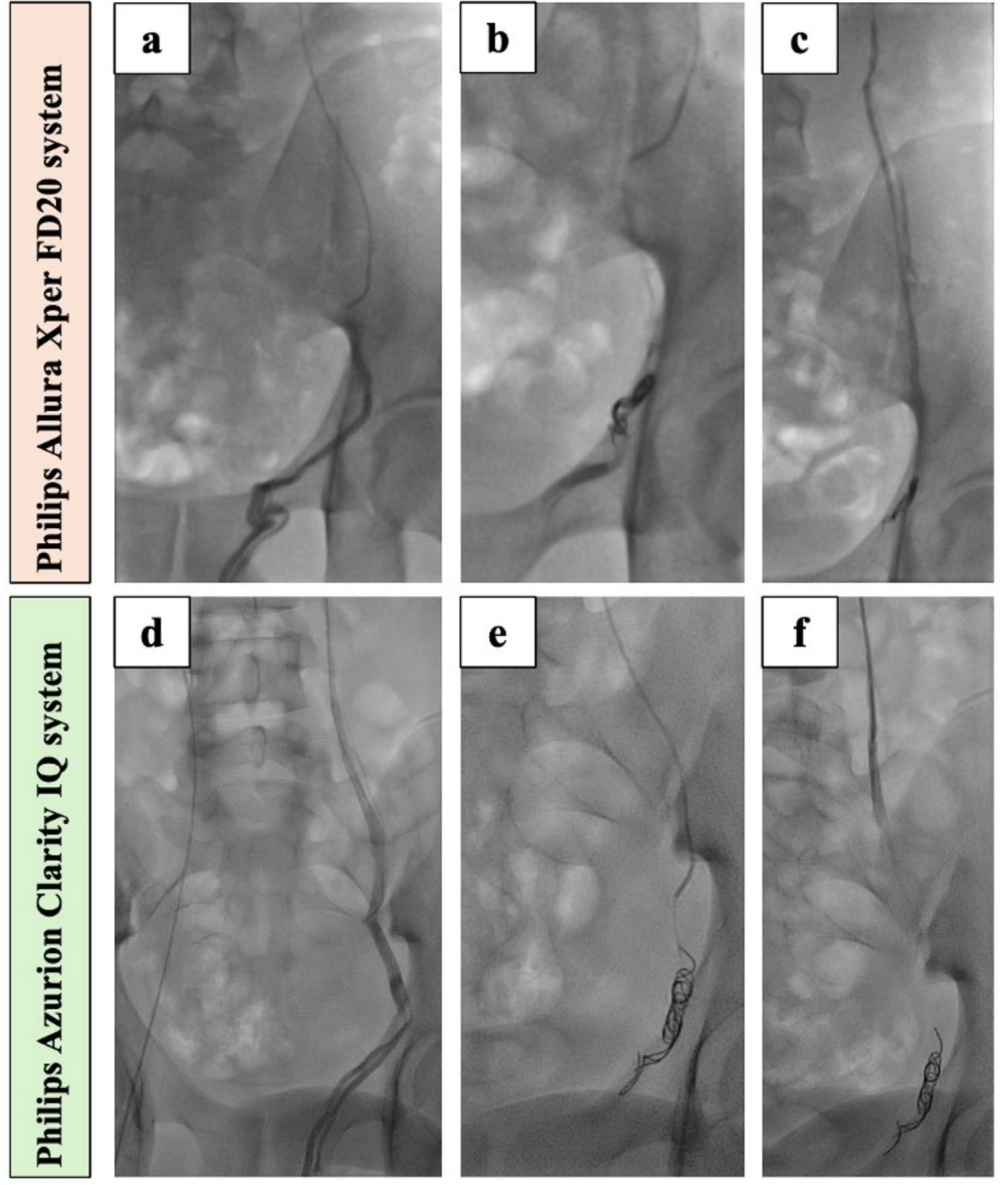
Comparison of image quality of Philips Allura Xper FD20 system (**a**, **b**, **c**) and Philips Azurion Clarity IQ system (**d**, **e**, **f**) during percutaneous varicocele embolization (PVE). (**a**) and (**d**) show spermatic vein course, size, collateral pathways, type and variations obtained with a firm manual injection of 5–10 mL of contrast medium during Valsalva maneuver. (**b**) and (**e**) show spermatic vein embolization with pushable 0.018’’ platinum microcoils. (**c**) and (**f**) show final contrast injection documenting spermatic vein distal occlusion after the injection of 2% Aethoxysklerol. Two blinded interventionalists agreed for a better image quality in Philips Azurion Clarity IQ technology

The overall image quality was superior in the procedures performed with the Philips Azurion system using Clarity IQ technology, as illustrated in Fig. 2. No significant inter-observer variability was recorded.

In pediatric subgroups (< 18 yrs), a statistically significant reduction in both values of DAP and Ka.ref was observed in subgroup 1 compared to subgroup 0: 13,153 vs 3880.75 mGy·cm^2^ (*p* < 0.001) and 52.77 vs 16.1 mGy (*p* < 0.001), respectively. Both the pediatric subgroups showed comparable values of procedural time (25 vs. 27.5 min for group 1 and group 0, respectively) as well as fluoro time (364.25 vs. 545 min for group 1 and group 0, respectively).

A statistically significant reduction of DAP and Ka.ref was observed in high-grade varicocele subgroup treated with System 1, with DAP values of 19,629 vs. 4028 mGy·cm^2^ (*p* < 0.001) and reference air kerma values of 73.5 vs. 19 mGy (*p* < 0.001). Furthermore, both subgroups revealed comparable values of procedural time (26.5 vs. 27 min for group 0 and group 1, respectively) and fluoro time (427.5 vs. 462 min for group 0 and group 1, respectively). The results of subpopulation analyses are summarized in Table 4.

**Table 4:** Summary of age and dosimetric parameters in patient subpopulations stratified by age (< 18 years) and by varicocele grade (Sarteschi grades III/IV)

Pediatric population	Group 0	Group 1	*p*-value
	*n* = 10	*n* = 24	
*Age (years)*			
Median	13.5	17	< 0.001
IQR	13–24	16–17.75	
*Fluoro time (sec)*			
Median	364.25	545	0.154
IQR	269.5–504	360–873.5	
*DAP (mGy·cm*^*2*^*)*			
Median	13,153	3880.75	< 0.001
IQR	10,310–17,773.5	1904–6182.5	
Ka.ref (mGy)			
Median	52.77	16.1	< 0.001
IQR	35.82–66.45	9.65–32.15	
*Procedure time (min)*			
Median	25	27.5	0.37
IQR	22.5–28	22.25–36.75	

Sarteschi grade III/IV	Group 0	Group 1	*p*-value
	*n* = 30	*n* = 45	
*Age*			
Median	24.5	18	0.244
IQR	16.75–29	17–26.5	
*Fluoro time (sec)*			
Median	427.5	462	0.057
IQR	296–567.25	363.5–853.5	
*DAP (mGy·cm*^*2*^*)*			
Median	19,629	4028	< 0.001
IQR	13,098–28,856.5	1861–6804	
*Ka.ref (mGy)*			
Median	73.5	19	< 0.001
IQR	52.35–104.4	11.7–34.75	
*Procedure time (min)*			
Median	26.5	27	0.541
IQR	23–30	21.5–36	

Group 0 refers to procedures performed using Philips Allura Xper FD20 system; group 1 refers to procedures performed using Philips Azurion Clarity IQ system

**Fig. 3:**
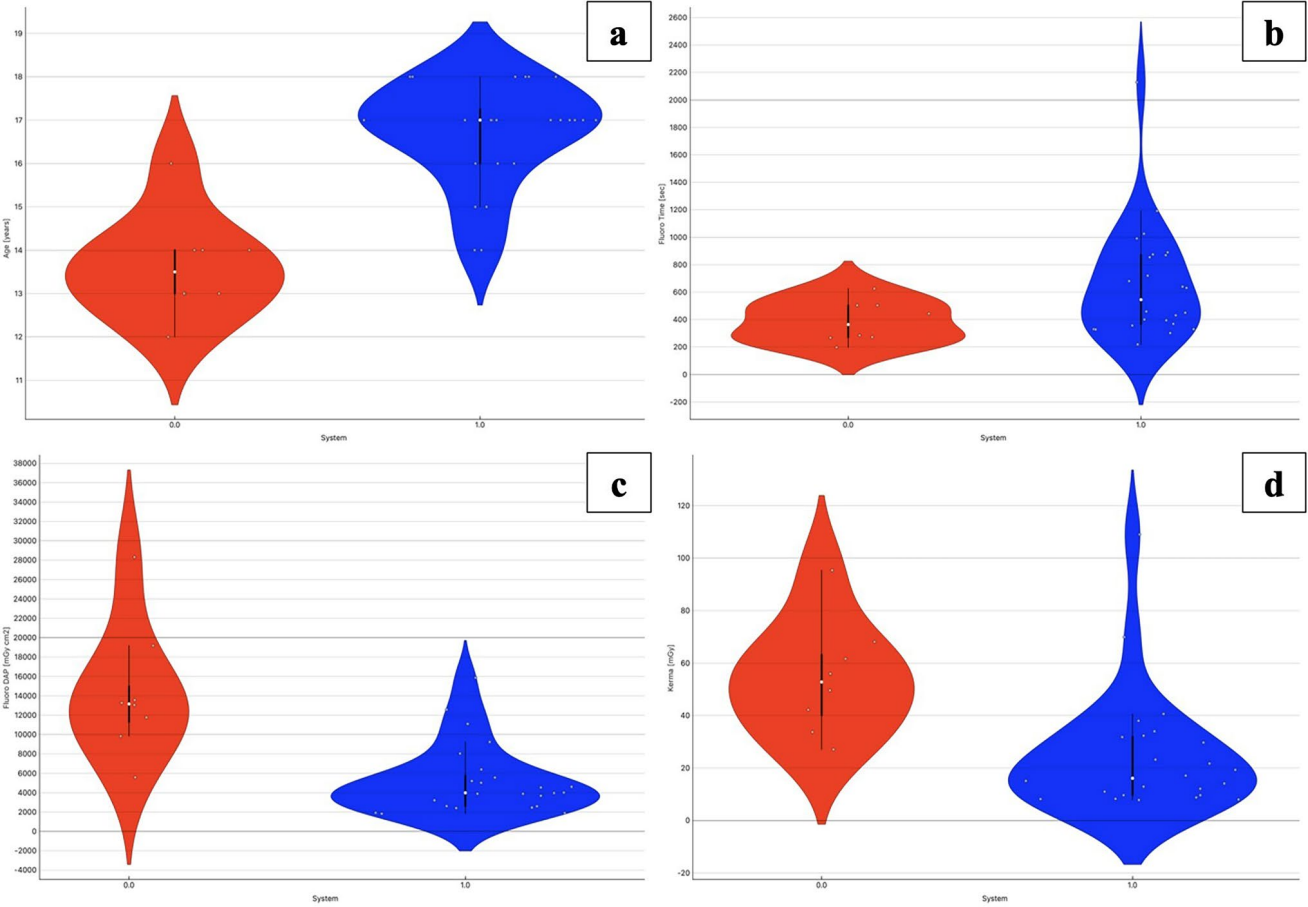
Violin plots comparing (**a**) fluoro time (s), (**b**) dose area product (DAP, mGy·cm^2^), and (**c**) reference air kerma (mGy) between System 0 (shown in red) and System 1 (shown in blue) in pediatric patient population. Each plot displays the distribution density, individual data points, median (white dot) and interquartile range (black bar). While fluoro time appeared higher and more variable with System 1, DAP and Kerma values were markedly lower, indicating improved radiation efficiency despite longer procedure durations. The plot dips below zero due to the smoothing kernel used in the violin plot reconstruction, which can interpolate values beyond the original data range, especially in regions with low data density

**Fig. 4:**
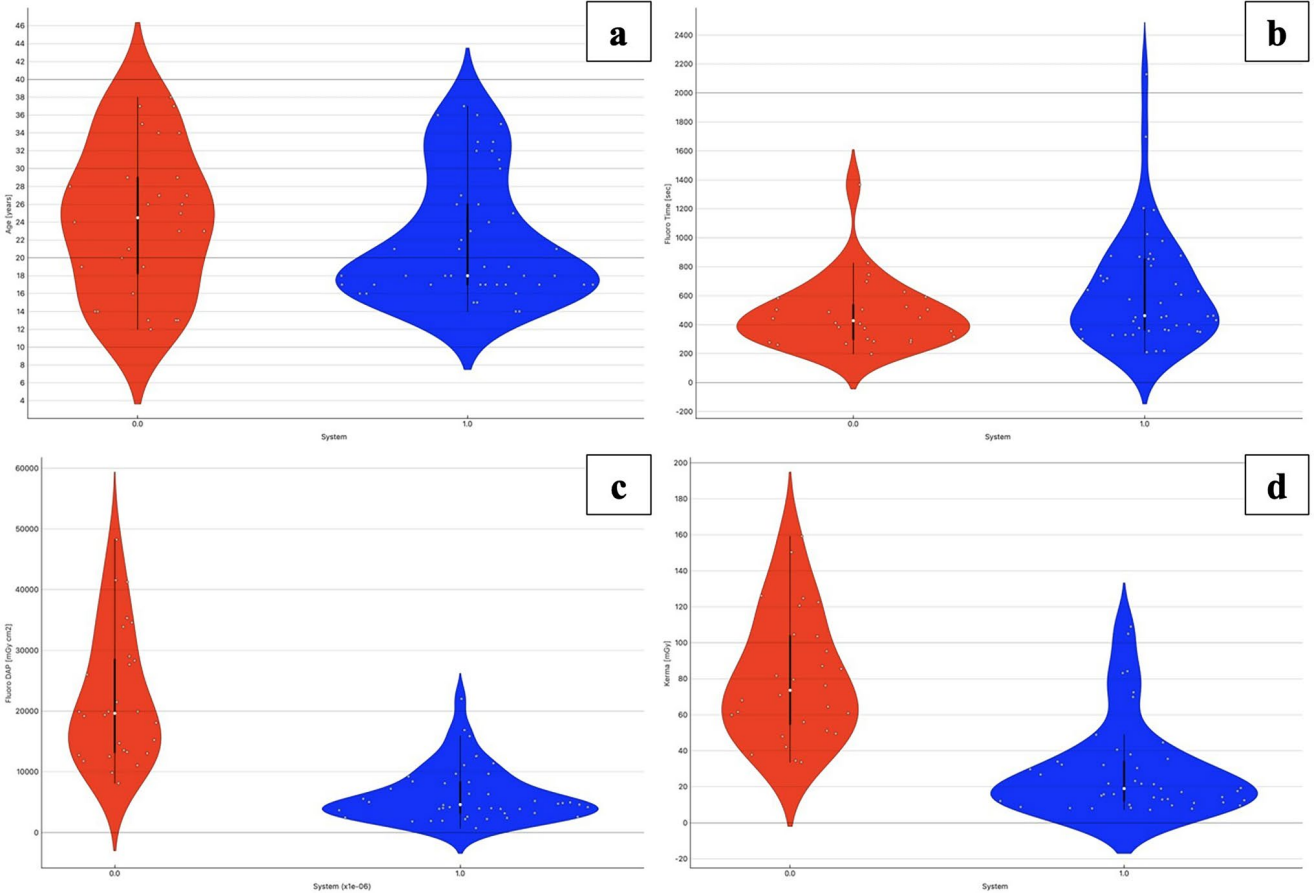
Violin plots comparing (**a**) fluoro time (s), (**b**) dose area product (DAP, mGy·cm^2^), and (**c**) reference air kerma (mGy) between System 0 (shown in red) and System 1 (shown in blue) in high-grade varicocele population. Each plot displays the distribution density, individual data points, median (white dot) and interquartile range (black bar). While fluoro time appeared higher and more variable with System 1, DAP and kerma values were markedly lower, indicating improved radiation efficiency despite longer procedure durations. The plot dips below zero due to the smoothing kernel used in the violin plot reconstruction, which can interpolate values beyond the original data range, especially in regions with low data density

Specifically, the Violin plots comparing pediatric subgroups in terms of procedure time, fluoro time, DAP and Ka.ref show a substantial narrowing of distribution and downward shift in dose-related values in the subgroup treated with System 1 (Fig. 3). Similarly, the high-grade varicocele subgroup (Fig. 4), treated with System 1, exhibited markedly lower DAP and reference air kerma values.

The 75th percentile of dose area product (DAP) for procedures performed with the Clarity IQ system (Group 1) was 8.06 Gy·cm^2^, which may serve as a preliminary procedure-specific diagnostic reference level (DRL) for male varicocele embolization.

## Discussion

Percutaneous endovascular embolization of ISV has been widely recognized as a safe and effective treatment for painful male varicocele and as a valid option to improve fertility in couples with abnormal sperm parameters [20–24]. In male varicocele treatment, PVE is nowadays preferred over traditional surgery, due to advantages in terms of minimal invasiveness, faster recovery and lower complication rates [25–27].

Despite its well-documented clinical efficacy, PVE involves exposure to ionizing radiation. This may represent a non-negligible drawback, especially in younger, otherwise healthy and radiosensitive patients [8]. Utmost attention to radiation dose is therefore a key concern to minimize long-term radiation-related risks in this patient population.

Our retrospective study aimed to compare radiation dose parameters obtained during PVE procedures performed using two successive generations of digital angiographic technology between 2020 and 2024.

The comparative analysis between the previously available Philips Allura Xper FD20 system (group 0) and the recently installed Philips Azurion Clarity IQ system (group 1) demonstrated a significant reduction in radiation exposure with the newer technology (group 1), demonstrating a 71.15% decrease in DAP and a 64.41% decrease in Ka.ref, independently of procedure duration and fluoro time. Notably, group 1 included younger patients and more complex varicocele cases and showed a broader distribution of fluoroscopy time, with some procedures lasting longer than in Group 0. Significantly lower dose values were obtained with Clarity IQ technology in slightly more complex scenarios, such as high-grade varicoceles or anatomical variants, which may require multiple and more technically challenging catheterizations. In accordance with these findings, in the subgroups dose analysis, system 1 demonstrated a superior performance compared to system 0, achieving a significantly noteworthy dose reduction in both the pediatric and the high-grade varicocele subgroups. This suggests high dosimetric benefits with system 1 for high-grade varicocele treatment and for pediatric patients. In fact, PVE is frequently performed in males with grade III and IV varicocele, who typically present with more severe venous dilation and challenging venous anatomy, leading to longer procedure and fluoroscopy times with higher radiation exposure [28]. Instead, pediatric patients as well as adolescents are both characterized by higher radiosensitivity, longer life expectancy, and increased risks of delayed radiation effects, and radiation-induced malignancies [28–30].

Despite the consistent dose reduction, a qualitative evaluation of the overall image quality based on three parameters suggested by previous studies demonstrated a better subjective perception of image quality obtained by Clarity IQ technology [21, 30, 31]. This qualitative evaluation suggests that dose reduction with newer optimization technologies does not compromise, but rather improves, the perceived image quality during and after the procedure.

In medical imaging, DRLs have proven to be a valuable tool for optimizing patient radiation exposure during radiological procedures. Over time, their relevance in interventional radiology has significantly increased and current European guidelines for radiation protection are being updated to include the interventional procedures most frequently performed in clinical routine [18, 32–34]. Despite this progress, several issues remain unsolved regarding both the methodology for establishing DRLs and their practical application in clinical settings [34]. This is particularly relevant in interventional procedures, where patient effective doses can vary considerably depending on the type of procedure, the operator’s experience, and the overall procedural complexity [11, 17]. To reduce this variability, the study population included only left-sided varicocele, treated by the same interventional radiology team, using the same right femoral vein access [35–37]. A standardized protocol was adopted to minimize radiation exposure and optimize image quality. Phlebographic sequences were deliberately limited to strictly necessary cases, and those cases were excluded from analysis. Additional strategies included the preference for lower pulse rates, maximum use of collimation, last-image-hold utilization, and minimized fluoroscopy time [8]. Ultimately, the operator’s dose awareness, supported by specific training in radiation dose management, was essential in applying these strategies and reducing exposure for both patients and staff [11].

One of the methods commonly used to establish DRLs is based on collecting data from a group of patients representative of general population and calculating the 75th percentile of the resulting dose distribution [15, 16]. This value may be considered as the DRL for a specific population undergoing a specific radiological examination. Within this framework, many European countries have already established national DRLs for certain interventional procedures [38–40]. However, PVE is not yet among them, despite the high radiosensitivity of the typical patient population [33].

Our findings may therefore support the introduction of PVE-related DRLs and encourage the adoption of low-dose angiographic systems in routine clinical practice. Based on data acquired, the 75th percentile DAP of 8.06 Gy·cm^2^ (Group 1) could serve as a preliminary reference value, derived from the system optimized for lower radiation exposure. This proposal is intended as a guideline while acknowledging the limitations of our dataset. As first, the monocentric and retrospective study design. Secondly, the long study period, during which treatment indications have constantly evolved, especially for younger patients. In recent years, a growing attention from pediatric and pediatric urology societies has contributed to an earlier varicocele treatment, aiming to preserve future fertility [4, 41]. According to the manufacturer and given the design of Clarity IQ technology, patients' body mass index (BMI) was not included as a variable.

Since the system works by adjusting the radiation output based on tissue attenuation, anatomical region, and image content, including BMI would not have significantly impacted the results related to dosimetric reduction achieved with this technology. The lack of BMI data in the present study still represents a potential limitation, as patient habitus may influence image acquisition parameters and contribute to variability in radiation exposure [32]. Therefore, future studies evaluating BMI and other habitus-related factors are advisable to further validate our results. Lastly, the absence of a quantitative analysis comparing the image quality of the angiographic systems represents a limitation to be addressed in future studies.

In conclusion, the integration of advanced dose-optimization technologies, together with standardized protocols and appropriate operator training, provides a key advantage in reducing dose exposure of young higher-risk patients, without compromising image quality, procedural efficiency, or clinical outcomes. A tailored dose-reduction strategy for male varicocele, combined with the adoption of advanced angiographic technologies may provide the framework for the introduction of procedure-related DRLs. However, these results are based on a single-center, retrospective cohort and should be validated by multicenter studies to ensure broader applicability and alignment with contemporary radiation protection standards.
